# From pre-DP, post-DP, SP4, and SP8 Thymocyte Cell Counts to a Dynamical Model of Cortical and Medullary Selection

**DOI:** 10.3389/fimmu.2014.00019

**Published:** 2014-02-14

**Authors:** Maria Sawicka, Gretta L. Stritesky, Joseph Reynolds, Niloufar Abourashchi, Grant Lythe, Carmen Molina-París, Kristin A. Hogquist

**Affiliations:** ^1^Department of Applied Mathematics, School of Mathematics, University of Leeds, Leeds, UK; ^2^Department of Laboratory Medicine and Pathology, Center for Immunology, University of Minnesota, Minneapolis, MN, USA

**Keywords:** thymocytes, negative selection, positive selection, death by neglect, mathematical model, steady state

## Abstract

Cells of the mature αβ T cell repertoire arise from the development in the thymus of bone marrow precursors (thymocytes). *αβ* T cell maturation is characterized by the expression of thousands of copies of identical *αβ* T cell receptors and the CD4 and/or CD8 co-receptors on the surface of thymocytes. The maturation stages of a thymocyte are: (1) double negative (DN) (TCR^−^, CD4^−^ and CD8^−^), (2) double positive (DP) (TCR^+^, CD4^+^ and CD8^+^), and (3) single positive (SP) (TCR^+^, CD4^+^ or CD8^+^). Thymic antigen presenting cells provide the appropriate micro-architecture for the maturation of thymocytes, which “sense” the signaling environment via their randomly generated TCRs. Thymic development is characterized by (i) an extremely low success rate, and (ii) the selection of a functional and self-tolerant T cell repertoire. In this paper, we combine recent experimental data and mathematical modeling to study the selection events that take place in the thymus after the DN stage. The stable steady state of the model for the pre-DP, post-DP, and SP populations is identified with the experimentally measured cell counts from 5.5- to 17-week-old mice. We make use of residence times in the cortex and the medulla for the different populations, as well as recently reported asymmetric death rates for CD4 and CD8 SP thymocytes. We estimate that 65.8% of pre-DP thymocytes undergo death by neglect. In the post-DP compartment, 91.7% undergo death by negative selection, 4.7% become CD4 SP, and 3.6% become CD8 SP. Death by negative selection in the medulla removes 8.6% of CD4 SP and 32.1% of CD8 SP thymocytes. Approximately 46.3% of CD4 SP and 27% of CD8 SP thymocytes divide before dying or exiting the thymus.

## 1 Introduction

T cells are a major component of the adaptive immune system that play a crucial role in protection against a wide variety of pathogens. The T cell receptor (TCR) is generated by somatic recombination and has a vast potential to recognize foreign organisms. T cells do not recognize pathogens directly, but rather through binding pathogen fragments displayed by major histocompatibility complex (MHC) proteins on the surface of antigen presenting cells (APCs). Since MHC molecules are highly polymorphic, useful T cells must be selected for in each individual of the species. These T cells must have lineage specific effector functions that may include direct lysis, production of cytokines, and ability to regulate immune responses. Furthermore, some T cells have the potential to drive dangerous autoimmune responses (1). For all of these reasons, the development of a T cell repertoire is a highly specialized and tightly regulated process (2, 3). It takes place in a dedicated organ, the thymus, where unique properties of the microenvironment ensure the production of functional, yet self-tolerant T cells (4–6).

Multi-potent precursors travel from the bone marrow to the thymus through the blood. When they enter the thymus, the precursors that commit to the T cell lineage [or canonical early T cell progenitors (7)], after a 2 week period on average, transition from the double negative (DN) stage, where they do not express the co-receptors CD4 and CD8, to the double positive (DP) stage, where they express both co-receptors (6). At this stage, the majority of the cells have made productive TCR gene rearrangements and express a fully formed *αβ* TCR on the cell surface. DP cells are located in the cortex region of the thymus, where they use their TCR to survey self-peptides presented by MHCs on cortical thymic epithelial cells (cTECs) (8). DPs that recognize self-peptide-MHC complexes with low affinity undergo positive selection, whereas those with high affinity are deleted by negative selection (3). Those DP that fail to recognize self-peptide-MHC will undergo apoptosis in a process referred to as death by neglect. The DP cells that are positively selected will then transition to the single positive (SP) stage, where they express either the CD4 or CD8 co-receptor, depending upon their MHC class specificity (9). MHC class specificity also dictates gene expression changes that will ultimately determine the effector functions of that T cell: generally, cytotoxicity for CD8 T cells and cytokine production for CD4 T cells. All positively selected cells, whether MHC class I or class II specific, up-regulate the chemokine receptor CCR7, which facilitates their migration to the medulla, where they undergo further selection events. The medulla contains medullary epithelial cells (mTECs) that express tissue-restricted antigens regulated by the nuclear factor Aire (10). Exposure to tissue-restricted antigens allows for further deletion of T cells specific for self-antigens they may encounter in the periphery. Finally, those cells that have been positively selected, yet have avoided negative selection, will mature and migrate to the periphery (11).

Previous efforts to develop mathematical models of thymic selection have been based on deterministic approaches or cellular automata simulations. These studies have shown the importance of (i) thymic antigen diversity on the size of the selected T cell repertoire (12), (ii) death rates for the more differentiated thymocyte subsets (13), (iii) thymocyte proliferation and residence times (14), (iv) epithelial networks for thymocyte development and migration (15), (v) thymocyte competition for antigen (16), (vi) self-pMHC complexes expressed on dendritic cells (DCs) (17), (vii) receptor–ligand binding affinity (18), and (viii) a sharp threshold in TCR-ligand binding affinity that defines the boundary between negative and positive selection (19). Recent work by Ribeiro and Perelson (20) supports the need to develop appropriate mathematical models to interpret T cell receptor excision circles (or TREC) data, which are used to quantify thymic export (20). Sinclair et al. in Ref. (21) bring together experimental immunology with mathematical modeling to conclude that CD8 precursor thymocytes are more susceptible to death than CD4 precursors. This asymmetry in the death rates underlies the experimentally observed CD4:CD8 T cell ratio in the periphery.

Previous experimental studies have tried to determine the number of cells going through positive and negative selection in the thymus. However, reports estimating the relative number of cells undergoing negative selection compared to positive selection have been widely variable. Some find that very few cells undergo negative selection; others find that two times more cells undergo negative selection than positive selection (22–25). In this report, we develop a deterministic mathematical model of T cell development in the thymus. Some of us recently published a report where we used a novel approach (Bim^−/−^Nur77^GFP^ mice) that allowed us to calculate the number of cells undergoing positive and negative selection (26). Using previously published data on the relative life-span of DP and SP cells, we estimated the hourly rate of both positive and negative selection (26). In this manuscript, we make use of (i) a subset of this experimental data, and (ii) the asymmetric death rates observed for CD4 and CD8 precursor thymocytes (21), to develop two mathematical models that will enable us to estimate selection rates in the cortex and the medulla, and provide a quantitative measure for the stringency of thymic selection. The first model (see Section 2.1) allows the identification of the following parameters: DN thymocyte influx into the cortex, pre-DP and post-DP death rates, and pre-DP and post-DP differentiation rates. Under the assumption of asymmetric death rates for the CD4 and CD8 SP thymocytes (21), we extend the first model to provide estimates for the following medullary rates (see Section 2.2): CD4 and CD8 SP death, proliferation, and maturation rates.

## 2 Materials and Methods

### 2.1 A first model of thymic development after the DN stage

In this section, we introduce a deterministic model of thymocyte development after the DN stage. This first model will be required to calibrate the parameter values of the second model introduced in Section 2.2. In particular, and as described in Section 3.2, the first model allows the identification of parameter values for the following rates: *ϕ, μ*_1_, *μ*_2_, *φ*_1_, and *φ*_2_.

This mathematical model makes use of a data set obtained from the analysis of eight C57BL/6 wild type and Bim deficient mice (average age 9 weeks), that express a Nur77^GFP^ transgene to indicate TCR signal strength experience (26). Flow cytometric analysis, as described in that study, used standard markers to define various stages of T cell development in the thymus. The Nur77^GFP^ reporter and Bim deficiency were novel modifications that allowed us to quantify cells that normally would be deleted by strong TCR signaling. In the mathematical model, we consider the following thymocyte populations: *n*_1_, the population of pre-selection DP thymocytes (double positive), that are TCR*β*^low^ and CD69^low^ (26), *n*_2_, the population of post-selection DP thymocytes, that are TCR*β*^+^ and CD69^high^ (26), and *n*_3_, the population of mature SP (single positive) thymocytes.

**Figure 1:**
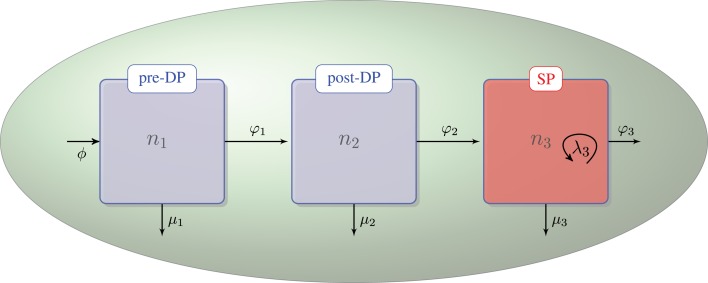
**Thymic development as hypothesized in the first model**. The flux, *ϕ*, represents the differentiation of DNs into pre-DPs. Pre-DP thymocytes have two fates: further differentiation into the post-DP pool (*φ*_1_) or death by neglect (*μ*_1_). Post-DP thymocytes have two fates: further differentiation into the SP pool (*φ*_2_) or death by apoptosis (*μ*_2_). Finally, SP thymocytes have three fates: maturation and exit into the periphery (*φ*_3_), death by apoptosis (*μ*_3_), or proliferation (*λ*_3_).

We assume that DN thymocytes differentiate to become pre-selection DP thymocytes with rate (cells/day) *ϕ*. We further assume that after the DN stage, thymocyte cell fate is determined by the TCR signal, which a given thymocyte has received. Sinclair et al. used CFSE labeling to show that there is no proliferation at the post-DP stage (see Figure A1 of their manuscript) (21). Stritesky et al. looked at proliferation in the post-DP pool with BrdU labeling, and found no evidence (26). We have, thus, only included proliferation in the SP thymocyte population (21, 26). The three populations, *n*_1_, *n*_2_, and *n*_3_, are involved in the following selection events in the cortex and the medulla (see Figure 1):

- $\emptyset \overset{\phi }{\to }n_{1}$ – flux of DN thymocytes into compartment *n*_1_,
- $n_{1}\overset{\varphi_{1}}{\to }n_{2}$ – differentiation from pre-DP (*n*_1_) to post-DP (*n*_2_) thymocytes induced by TCR signal,
- $n_{1}\overset{\mu_{1}}{\to }\emptyset$ – death by neglect of pre-DP thymocytes due to lack of (or weak) TCR signal,
- $n_{2}\overset{\varphi_{2}}{\to }n_{3}$ – differentiation from post-DP (*n*_2_) to SP (*n*_3_) thymocytes sustained by intermediate TCR signal,
- $n_{2}\overset{\mu_{2}}{\to }\emptyset$ – apoptosis of post-DP (*n*_2_) thymocytes due to strong TCR signal,
- $n_{3}\overset{\varphi_{3}}{\to }\mathrm{periphery}$ – exit of SP thymocytes (*n*_3_) to the periphery (thymic maturation),
- $n_{3}\overset{\lambda_{3}}{\to }2\;n_{3}$ – proliferation of SP thymocytes (*n*_3_) in the medulla, and
- $n_{3}\overset{\mu_{3}}{\to }\emptyset$ – apoptosis of SP (*n*_3_) thymocytes due to strong TCR signal.

The time evolution of the three populations can be described by the following set of ordinary differential equations (ODEs), which are based on the selection events described above:

(1) $$\begin{array}{c} \left\{\begin{array}{c} \frac{dn_{1}}{dt}=\phi -\varphi_{1}n_{1}-\mu_{1}n_{1}\;,\;\\ \frac{dn_{2}}{dt}=\varphi_{1}n_{1}-\varphi_{2}n_{2}-\mu_{2}n_{2}\;,\;\\ \frac{dn_{3}}{dt}=\varphi_{2}n_{2}-\varphi_{3}n_{3}-\mu_{3}n_{3}+\lambda_{3}n_{3}\;.\; \end{array}\right. \end{array}$$

We are interested in studying the steady state of these populations, as the experimental data correspond to population cell numbers in the three stages (pre-DP, post-DP, and SP) for a steady state thymus (26). The steady state of the system of equations [equation (1)] is given by:

(2) $$n_{1}^{*}=\frac{\phi }{\varphi_{1}+\mu_{1}}\;,\;n_{2}^{*}=\frac{n_{1}^{*}\varphi_{1}}{\varphi_{2}+\mu_{2}}\;,\;n_{3}^{*}=\frac{n_{2}^{*}\varphi_{2}}{\varphi_{3}+\mu_{3}-\lambda_{3}}\;.$$

This unique steady state exists if and only if *φ*_3_ + *μ*_3_ − *λ*_3_ > 0, so that we have $n_{3}^{*}>0$. In order to study the linear stability of the steady state, we calculate *A*, the Jacobian matrix of equation (1), as follows:

(3) $$A=\left(\begin{array}{ccc} -\left(\varphi_{1}+\mu_{1}\right) & 0 & 0\\ \varphi_{1} & -\left(\varphi_{2}+\mu_{2}\right) & 0\\ 0 & \varphi_{2} & -\left(\varphi_{3}+\mu_{3}-\lambda_{3}\right) \end{array}\right).$$

*A* is also the Jacobian matrix at the steady state $n^{*}=\left(n_{1}^{*},n_{2}^{*},n_{3}^{*}\right)$, as the system of ODEs [equation (1)] is linear. The three eigenvalues of *A* are given by (as the matrix is lower triangular):

$$\beta_{1}=-\left(\varphi_{1}+\mu_{1}\right)\;,\;\beta_{2}=-\left(\varphi_{2}+\mu_{2}\right)\;,\;\beta_{3}=-\left(\varphi_{3}+\mu_{3}-\lambda_{3}\right)\;.$$

Therefore, the steady state [equation (2)] is stable, if and only if, *φ*_3_ + *μ*_3_ − *λ*_3_ > 0, which is also the condition for its existence. We conclude this section with the analytical solution of the system of ODEs [equation (1)], given initial conditions, which provides the time evolution of the three thymocyte populations:

(4) $$\begin{array}{llll} n_{1}\left(t\right) & =n_{1}^{*}+n_{1}\left(0\right)\;e^{-\left(\varphi_{1}+\mu_{1}\right)t}\;,\; & & \;\\ n_{2}\left(t\right) & =n_{2}^{*}+\frac{n_{1}\left(0\right)\;\varphi_{1}}{\left[\left(\varphi_{2}+\mu_{2}\right)-\left(\varphi_{1}+\mu_{1}\right)\right]}\;e^{-\left(\varphi_{1}+\mu_{1}\right)t}\; & & \;\\ & \;+n_{2}\left(0\right)\;e^{-\left(\varphi_{2}+\mu_{2}\right)t}\;,\; & & \;\\ n_{3}\left(t\right) & =n_{3}^{*}+\frac{n_{1}\left(0\right)\;\varphi_{1}}{\left[\left(\varphi_{2}+\mu_{2}\right)-\left(\varphi_{1}+\mu_{1}\right)\right]}\\ & \;\times \frac{\varphi_{2}}{\left[\left(\varphi_{3}+\mu_{3}-\lambda_{3}\right)-\left(\varphi_{1}+\mu_{1}\right)\right]}\;e^{-\left(\varphi_{1}+\mu_{1}\right)t}\; & & \;\\ & \;+\frac{n_{2}\left(0\right)\;\varphi_{3}}{\left[\left(\varphi_{3}+\mu_{3}-\lambda_{3}\right)-\left(\varphi_{2}+\mu_{2}\right)\right]}\;e^{-\left(\varphi_{2}+\mu_{2}\right)t}\; & & \;\\ & \;+n_{3}\left(0\right)\;e^{-\left(\varphi_{3}+\mu_{3}-\lambda_{3}\right)t}\;,\; & & \; \end{array}$$

where *n*_1_(0), *n*_2_(0), *n*_3_(0) represent the initial conditions for the thymocyte populations. Note that in the late time limit, that is, if *t* → +∞ and *φ*_3_ + *μ*_3_ − *λ*_3_ > 0, then $n_{1}\left(t\right)\to n_{1}^{*},n_{2}\left(t\right)\to n_{2}^{*}$ and $n_{3}\left(t\right)\to n_{3}^{*}$, as it is the unique stable steady state.

### 2.2 A second model of thymic development after the DN stage: CD4 and CD8 SP thymocytes

As described in Section 2.1, the first deterministic model will allow us to calibrate some of the parameters of a more comprehensive model, which we now introduce. We subdivide the SP thymocyte population in two classes: CD4 SP and CD8 SP thymocytes. This is an extension of the model introduced in the previous section, and is motivated by the fact that experimentally, SP thymocytes express either the CD4 or the CD8 co-receptor. We now have four different thymocyte populations to consider: *n*_1_, the population of pre-selection DP (double positive) thymocytes, *n*_2_, the population of post-selection DP thymocytes, *n*_4_, the population of mature CD4^+^ SP (single positive) thymocytes, and *n*_8_, the population of mature CD8^+^ SP (single positive) thymocytes.

**Figure 2:**
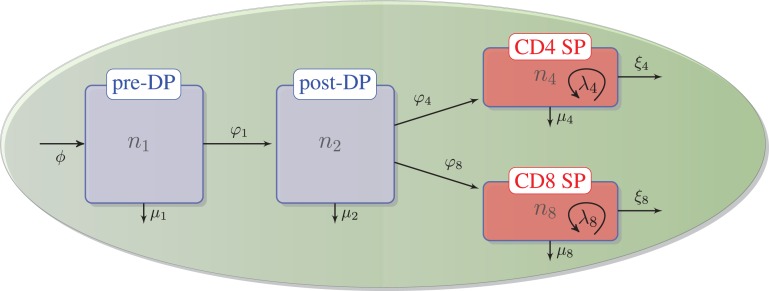
**Thymic development as hypothesized in the second model**. We assume there is a flux, *ϕ*, that represents the differentiation of DNs into pre-DPs. Pre-DP thymocytes have two fates: further differentiation into the post-DP pool (*φ*_1_) or death by neglect (*μ*_1_). Post-DP thymocytes have three fates: further differentiation into the SP pool as CD4 SPs (*φ*_4_), or CD8 SPs (*φ*_8_), or death by apoptosis (*μ*_2_). Finally, CD4 or CD8 SP thymocytes have three fates: maturation and exit into the periphery (*ξ*_4_) or (*ξ*_8_), death by apoptosis (*μ*_4_) or (*μ*_8_), or proliferation (*λ*_4_) or (*λ*_8_).

As described in the previous section, we assume that DN thymocytes differentiate to become pre-selection DP thymocytes with rate (cells/day) *ϕ*, and that after the DN stage, thymocyte cell fate is determined by the TCR signal, which a given thymocyte has received. Thus, the four populations, *n*_1_, *n*_2_, *n*_4_, and *n*_8_, with *n*_3_ = *n*_4_ + *n*_8_, are involved in the following selection events in the cortex and the medulla (see Figure 2):

- $\emptyset \overset{\phi }{\to }n_{1}$ – flux of DN thymocytes into compartment *n*_1_,
- $n_{1}\overset{\varphi_{1}}{\to }n_{2}$ – differentiation from pre-DP (*n*_1_) to post-DP (*n*_2_) thymocytes induced by TCR signal,
- $n_{1}\overset{\mu_{1}}{\to }\emptyset$ – death by neglect of pre-DP thymocytes due to lack of (or weak) TCR signal,
- $n_{2}\overset{\varphi_{4}}{\to }n_{4}$ – differentiation from post-DP (*n*_2_) to CD4^+^ SP (*n*_4_) sustained by intermediate TCR signal,
- $n_{2}\overset{\varphi_{8}}{\to }n_{8}$ – differentiation from post-DP (*n*_2_) to CD8^+^ SP (*n*_8_) sustained by intermediate TCR signal,
- $n_{2}\overset{\mu_{2}}{\to }\emptyset$ – apoptosis of post-DP (*n*_2_) thymocytes due to strong TCR signal,
- $n_{4}\overset{\xi_{4}}{\to }\mathrm{periphery}$ – exit of CD4^+^ SP thymocytes (*n*_4_) to the periphery (thymic maturation),
- $n_{8}\overset{\xi_{8}}{\to }\mathrm{periphery}$ – exit of CD8^+^ SP thymocytes (*n*_8_) to the periphery (thymic maturation),
- $n_{4}\overset{\lambda_{4}}{\to }2\;n_{4}$ – proliferation of CD4^+^ SP thymocytes (*n*_4_) in the medulla,
- $n_{8}\overset{\lambda_{8}}{\to }2\;n_{8}$ – proliferation of CD8^+^ SP thymocytes (*n*_8_) in the medulla,
- $n_{4}\overset{\mu_{4}}{\to }\emptyset$ – apoptosis of CD4^+^SP (*n*_4_) thymocytes due to strong TCR signal, and
- $n_{8}\overset{\mu_{8}}{\to }\emptyset$ – apoptosis of CD8^+^SP (*n*_8_) thymocytes due to strong TCR signal.

We assume that all model parameters are positive, that is, *μ*_1_, *μ*_2_, *μ*_4_, *μ*_8_, *φ*_1_, *φ*_2_, *φ*_4_, *φ*_8_, *ξ*_4_, *ξ*_8_, *ϕ, λ*_4_, *λ*_8_ > 0, and note that the parameters and thymocyte populations of the first and second model are related by the following equations:

(5) $$\begin{array}{llll} & \varphi_{2}=\varphi_{4}+\varphi_{8}\;,\;\xi_{4}\;n_{4}+\xi_{8}\;n_{8}=\varphi_{3}\;n_{3}\;,\; & & \;\\ & \;\mu_{4}\;n_{4}+\mu_{8}\;n_{8}=\mu_{3}\;n_{3}\;,\;\lambda_{4}\;n_{4}+\lambda_{8}\;n_{8}=\lambda_{3}\;n_{3}\;. \end{array}$$

The time evolution of the four populations can be described by the following set of ODEs:

(6) $$\begin{array}{c} \left\{\begin{array}{c} \frac{dn_{1}}{dt}=\phi -\varphi_{1}n_{1}-\mu_{1}n_{1}\;,\;\\ \frac{dn_{2}}{dt}=\varphi_{1}n_{1}-\left(\varphi_{4}+\varphi_{8}\right)n_{2}-\mu_{2}n_{2}\;,\;\\ \frac{dn_{4}}{dt}=\varphi_{4}n_{2}-\xi_{4}n_{4}-\mu_{4}n_{4}+\lambda_{4}n_{4}\;,\;\\ \frac{dn_{8}}{dt}=\varphi_{8}n_{2}-\xi_{8}n_{8}-\mu_{8}n_{8}+\lambda_{8}n_{8}\;.\; \end{array}\right. \end{array}$$

We are interested in studying the steady state of these populations, as the experimental data correspond to population cell numbers in the four stages (pre-DP, post-DP, CD4 SP, and CD8 SP) for a steady state thymus (26). The steady state of the system of equations [equation (6)] is given by:

(7) $$\begin{array}{llll} n_{1}^{*} & =\frac{\phi }{\varphi_{1}+\mu_{1}}\;,\;n_{2}^{*}=\frac{n_{1}^{*}\varphi_{1}}{\varphi_{4}+\varphi_{8}+\mu_{2}}\;,\; & & \;\\ n_{4}^{*} & =\frac{n_{2}^{*}\varphi_{4}}{\xi_{4}+\mu_{4}-\lambda_{4}}\;,\;n_{8}^{*}=\frac{n_{2}^{*}\varphi_{8}}{\xi_{8}+\mu_{8}-\lambda_{8}}\;.\; \end{array}$$

This unique steady state exists if and only if *ξ*_4_ + *μ*_4_ − *λ*_4_ > 0 and *ξ*_8_ + *μ*_8_ − *λ*_8_ > 0, so that we guarantee $n_{4}^{*}>0$ and $n_{8}^{*}>0$. In order to study the linear stability of the steady state, we calculate *B*, the Jacobian matrix of equation (6), as follows:

(8) $$B=\left(\begin{array}{cccc} -\left(\varphi_{1}+\mu_{1}\right) & 0 & 0 & 0\\ \varphi_{1} & -\left(\varphi_{4}+\varphi_{8}+\mu_{2}\right) & 0 & 0\\ 0 & \varphi_{4} & -\left(\xi_{4}+\mu_{4}-\lambda_{4}\right) & 0\\ 0 & \varphi_{8} & 0 & -\left(\xi_{8}+\mu_{8}-\lambda_{8}\right) \end{array}\right)\;.$$

*B* is also the Jacobian at the steady state $n^{*}=\left(n_{1}^{*},n_{2}^{*},n_{4}^{*},n_{8}^{*}\right)$, as the system of ODEs is linear. The four eigenvalues of *B* are given by:

$$\begin{array}{llll} & \beta_{1}=-\left(\varphi_{1}+\mu_{1}\right)\;,\;\beta_{2}=-\left(\varphi_{4}+\varphi_{8}+\mu_{2}\right)\;,\; & & \;\\ & \beta_{3}=-\left(\xi_{4}+\mu_{4}-\lambda_{4}\right)\;,\;\beta_{4}=-\left(\xi_{8}+\mu_{8}-\lambda_{8}\right)\;.\; & & \; \end{array}$$

Therefore, the steady state equation (6) is stable if and only if *ξ*_4_ + *μ*_4_ − *λ*_4_ > 0 and *ξ*_8_ + *μ*_8_ − *λ*_8_ > 0, which is also the condition for its existence. We conclude this section with the analytical solution of the system of ODEs equation (6), given initial conditions, which provides the time evolution of the four thymocyte populations:

(9) $$\begin{array}{llll} n_{1}\left(t\right) & =n_{1}^{*}+n_{1}\left(0\right)\;e^{-\left(\varphi_{1}+\mu_{1}\right)t}\;,\; & & \;\\ n_{2}\left(t\right) & =n_{2}^{*}+\frac{n_{1}\left(0\right)\;\varphi_{1}}{\left[\left(\varphi_{4}+\varphi_{8}+\mu_{2}\right)-\left(\varphi_{1}+\mu_{1}\right)\right]}\;e^{-\left(\varphi_{1}+\mu_{1}\right)t}\; & & \;\\ & \;+n_{2}\left(0\right)\;e^{-\left(\varphi_{4}+\varphi_{8}+\mu_{2}\right)t}\;,\; & & \;\\ n_{4}\left(t\right) & =n_{4}^{*}+\frac{n_{1}\left(0\right)\;\varphi_{1}}{\left[\left(\varphi_{4}+\varphi_{8}+\mu_{2}\right)-\left(\varphi_{1}+\mu_{1}\right)\right]} & \\ & \;\times \frac{\varphi_{4}+\varphi_{8}}{\left[\left(\xi_{4}+\mu_{4}-\lambda_{4}\right)-\left(\varphi_{1}+\mu_{1}\right)\right]}\;e^{-\left(\varphi_{1}+\mu_{1}\right)t}\\ & \;+\frac{n_{2}\left(0\right)\;\xi_{4}}{\left[\left(\xi_{4}+\mu_{4}-\lambda_{4}\right)-\left(\varphi_{4}+\varphi_{8}+\mu_{2}\right)\right]}\;e^{-\left(\varphi_{4}+\varphi_{8}+\mu_{2}\right)t}\\ & \;+n_{4}\left(0\right)\;e^{-\left(\xi_{4}+\mu_{4}-\lambda_{4}\right)t}\;,\\ n_{8}\left(t\right) & =n_{8}^{*}+\frac{n_{1}\left(0\right)\;\varphi_{1}}{\left[\left(\varphi_{4}+\varphi_{8}+\mu_{2}\right)-\left(\varphi_{1}+\mu_{1}\right)\right]}\\ & \;\times \frac{\varphi_{4}+\varphi_{8}}{\left[\left(\xi_{8}+\mu_{8}-\lambda_{8}\right)-\left(\varphi_{1}+\mu_{1}\right)\right]}\;e^{-\left(\varphi_{1}+\mu_{1}\right)t}\\ & \;+\frac{n_{2}\left(0\right)\;\xi_{8}}{\left[\left(\xi_{8}+\mu_{8}-\lambda_{8}\right)-\left(\varphi_{4}+\varphi_{8}+\mu_{2}\right)\right]}\;e^{-\left(\varphi_{4}+\varphi_{8}+\mu_{2}\right)t}\\ & \;+n_{8}\left(0\right)\;e^{-\left(\xi_{8}+\mu_{8}-\lambda_{8}\right)t}\;, \end{array}$$

where *n*_1_(0), *n*_2_(0), *n*_4_(0), *n*_8_(0) represent the initial conditions for the thymocyte populations. Note that in the late time limit, that is, if *t* → +∞ and *ξ*_4_ + *μ*_4_ − *λ*_4_ > 0 and *ξ*_8_ + *μ*_8_ − *λ*_8_ > 0, then $n_{1}\left(t\right)\to n_{1}^{*},n_{2}\left(t\right)\to n_{2}^{*}$, $n_{4}\left(t\right)\to n_{4}^{*}$, and $n_{8}\left(t\right)\to n_{8}^{*}$, as it is the unique stable steady state.

## 3 Results

### 3.1 Parameter estimation for the first model (means)

In this section, we make use of previously published experimental data (26) that provide thymocyte cell counts for the three subsets considered in the first model: pre-DPs, post-DPs, and SPs. The original experiments have been carried out for two types of mice: wild type mice (Bim^+/−^) and Bim deficient mice (Bim^−/−^). In this paper, we will only be considering the wild type experimental results. The data will allow us to provide experimental estimates for the steady state thymocyte cell counts: $n_{1}^{*},n_{2}^{*},n_{3}^{*}$. Note that, in order to estimate rates (with units of inverse time), thymocyte cell counts are not enough. Thus, we will make use of the additional knowledge provided by experimentally determined residence times for each population, *τ_i_*, with *i* = 1, 2, 3. If we make use of the model (see Section 2.1), the residence time in compartment *i* can be expressed as:

$$\tau_{i}=\frac{1}{\varphi_{i}+\mu_{i}}\;,\;\mathrm{for}\;i\in \left\{1,2,3\right\}\;.$$

The experimental data (see Table 1) correspond to the number of cells (thymocytes) at steady state (26), in each of the thymic compartments considered in the mathematical models (see Sections 2.1 and 2.2), and for eight different mice (*j* = 1, 2, …, 8).

**Table 1:** **Experimental steady state thymocyte cell counts for the wild type pre-DP, post-DP, CD4 SP, and CD8 SP populations**.

Mouse	$n_{1}^{*}$(pre-DP) (cells)	$n_{2}^{*}$(post-DP) (cells)	$n_{3}^{*}$(SP) (cells)	$n_{4}^{*}$(SP CD4) (cells)	$n_{8}^{*}$(SP CD8) (cells)
1	82.58 × 10^6^	9.30 × 10^6^	18.36 × 10^6^	13.85 × 10^6^	4.51 × 10^6^
2	142.19 × 10^6^	19.94 × 10^6^	26.20 × 10^6^	18.73 × 10^6^	7.46 × 10^6^
3	89.00 × 10^6^	5.98 × 10^6^	15.98 × 10^6^	11.88 × 10^6^	4.10 × 10^6^
4	29.32 × 10^6^	2.09 × 10^6^	5.61 × 10^6^	4.40 × 10^6^	1.21 × 10^6^
5	29.32 × 10^6^	2.09 × 10^6^	5.61 × 10^6^	4.40 × 10^6^	1.21 × 10^6^
6	51.26 × 10^6^	5.93 × 10^6^	9.01 × 10^6^	6.85 × 10^6^	2.16 × 10^6^
7	64.48 × 10^6^	6.81 × 10^6^	11.64 × 10^6^	9.03 × 10^6^	2.61 × 10^6^
8	218.94 × 10^6^	15.42 × 10^6^	40.20 × 10^6^	29.46 × 10^6^	10.74 × 10^6^
**Mean**	**88.39** × 10^6^	**8.45** × 10^6^	**16.57** × 10^6^	**12.33** × 10^6^	**4.25** × 10^6^
**Standard deviation**	**60.11** × 10^6^	**5.89** × 10^6^	**11.05** × 10^6^	**7.94** × 10^6^	**3.12** × 10^6^

*The bold font highlights the mean and the standard deviation from the individual mice data*.

We have made use of the following average residence times in each compartment (27–29)

$$\begin{array}{llll} & \tau_{1}=60\;h=2.5\;\mathrm{days}\;,\;\tau_{2}=16\;h=0.67\;\mathrm{days}\;,\; & & \;\\ & \tau_{3}=96\;h=4\;\mathrm{days}\;.\; & & \; \end{array}$$

**Figure 3:**
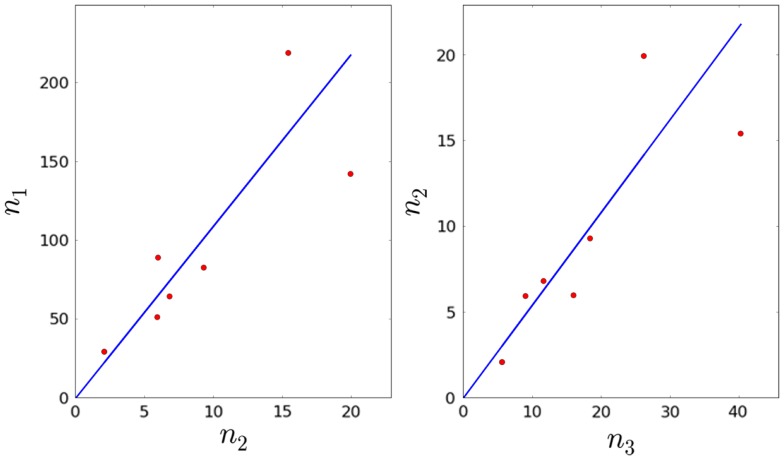
**Linear regression plots for the first model**.

In order to derive estimates for the model parameters, we have carried out the following steps:

1. We make use of the experimentally determined mature SP thymocyte flux from the medulla to the periphery, which has been estimated to be 1–4 × 10^6^ cells per day (14, 26, 30). This flux corresponds to about 1% of thymocytes leaving the thymus every day (30). Given this flux, which we denote by *ϕ*_out_, $n_{3}^{*}$, and the fact that $\phi_{\mathrm{out}}=\varphi_{3}\;n_{3}^{*}\;,$ we can obtain an estimate for *φ*_3_. We have chosen *ϕ*_out_ to be 2.5 × 10^6^ cells per day (14, 30).
2. Given *τ*_3_, *φ*_3_, and the fact that $\tau_{3}=\frac{1}{\mu_{3}+\varphi_{3}}\;,$ we can obtain an estimate for *μ*_3_.
3. Given *τ*_1_, $n_{1}^{*}$, and the fact that $n_{1}^{*}=\phi \;\tau_{1}\;,$ we can obtain an estimate for *ϕ*.
4. We also have $n_{2}^{*}=\varphi_{1}\;\tau_{2}\;n_{1}^{*}\;,$ which, in principle, allows us to estimate *φ*_1_. We make use of linear regression techniques to do so (31, 32).
  Let us introduce *a*_1_ by the following equation, $a_{1}=\frac{n_{2}^{*}}{n_{1}^{*}}$, and make use of the experimental data to write: $n_{2}^{*,i}=a\;n_{1}^{*,i}+\varepsilon^{i}$, for *i* = 1, 2, …, 8. Thus, the squared error is given by:
  $$E\left(a_{1}\right)=\sum_{i=1}^{8}\;{\left(n_{2}^{*,i}-a_{1}\;n_{1}^{*,i}\right)}^{2}\;.$$
  We minimize *E*(*a*_1_) with respect to *a*_1_, that is $\frac{dE}{da_{1}}=0$. Solving for *a*_1_, we obtain:
  $$a_{1}=\frac{\sum_{i=1}^{8}\;n_{1}^{*,i}\;n_{2}^{*,i}}{\sum_{i=1}^{8}\;{\left(n_{1}^{*,i}\right)}^{2}}\;.$$
  Given *a*_1_, we can then estimate *φ*_1_ from the equation $\varphi_{1}=\frac{a_{1}}{\tau_{2}}\;.$
5. Given *τ*_1_, *φ*_1_, and the fact that $\tau_{1}=\frac{1}{\mu_{1}+\varphi_{1}}\;,$ we can obtain an estimate for *μ*_1_.
6. We are now left with three remaining parameters: *φ*_2_, *μ*_2_, and *λ*_3_. Given the experimental constraints on *τ*_1_, *τ*_2_, and *τ*_3_, we assume that the average time to proliferate, 1/*λ*_3_, cannot be <7 days. Therefore, we consider *λ*_3_ to be constrained in the interval $\left[1∕7,\tau_{3}^{-1}\right]$, with time measured in days. We sample equally spaced values for *λ*_3_, and for each value, we compute $\varphi_{2}=\frac{n_{3}^{*}\left(\varphi_{3}+\mu_{3}-\lambda_{3}\right)}{n_{2}^{*}}$. The ratio $a_{2}=\frac{n_{3}^{*}}{n_{2}^{*}}$ is computed using the linear regression method described above (see Figure 3). In this way, we obtain an estimate for *φ*_3_. We note that the *p*-values for *a*_1_ and *a*_2_ are given by 7.57 × 10^−3^ and 6.85 × 10^−3^, respectively (both smaller than the significance level *α* = 0.05).
7. Given *τ*_2_, *φ*_2_, and the fact that $\tau_{2}=\frac{1}{\mu_{2}+\varphi_{2}}$, we can obtain an estimate for *μ*_2_.
8. From steps 6 and 7 above, we have generated (a table of) values for *φ*_2_ and *μ*_2_, given a fiducial value for λ_3_ in the interval $\left[1∕7,\tau_{3}^{-1}\right]$. The mice considered in the experimental study are 5.5–17 weeks old, and their thymus is in steady state (26). Thus, we expect that the parameter values can only be accepted if the corresponding system of ODEs attains steady state by 3 weeks. Therefore, we only accept parameter values which provide thymocyte cell counts at time *t* = 21 days that are within one standard deviation from the experimentally determined values (see Table 1). That is, we impose for the given parameter set that the mathematically predicted value *n_i_*(*t* = 21 days) belongs to the interval $n_{i}^{*}\pm \sigma_{i}$, with *i* = 1, 2, 3, and where $n_{i}^{*}$ is the (experimental) mean number of cells in compartment *i*, and *σ_i_* is the (experimental) standard deviation in compartment *i*, as given in Table 1.

We obtain the following parameter values:

$$\begin{array}{llll} & \phi =35.350\times 10^{6}\;\mathrm{cells}∕\mathrm{day}\;,\;\;\mu_{1}=0.263\;{\mathrm{day}}^{-1}\;,\; & & \;\\ & \mu_{3}=0.099\;{\mathrm{day}}^{-1}\;,\;\;\varphi_{1}=0.137\;{\mathrm{day}}^{-1}\;,\;\;\varphi_{3}=0.151\;{\mathrm{day}}^{-1}\;,\; & & \; \end{array}$$

and

$$\begin{array}{llll} & \mu_{2}\in \left[1.295,1.443\right]\;{\mathrm{day}}^{-1}\;,\;\;\varphi_{2}\in \left[0.050,0.198\right]\;{\mathrm{day}}^{-1}\;,\; & & \;\\ & \lambda_{3}\in \left[0.143,0.250\right]\;{\mathrm{day}}^{-1}\;.\; & & \; \end{array}$$

These parameters imply the following thymic selection rates:

#### 3.1.1 Death rates

9.7 × 10^5^ cells/h die by neglect in compartment 1 ($\mu_{1}\;n_{1}^{*}$), 4.8 × 10^5^ cells/h die by negative selection in compartment 2 ($\mu_{2}\;n_{2}^{*}$), and 6.9 × 10^4^ cells/h die by negative selection in compartment 3 ($\mu_{3}\;n_{3}^{*}$).

#### 3.1.2 Differentiation rates

5.0 × 10^5^ cells/h are positively selected in compartment 1, that is, become post-DP from pre-DP ($\varphi_{1}\;n_{1}^{*}$), 4.4 × 10^4^ cells/h are positively selected in compartment 2, that is, become SP from post-DP ($\varphi_{2}\;n_{2}^{*}$), and 1.0 × 10^5^ cells/h leave compartment 3 to go to the periphery ($\varphi_{3}\;n_{3}^{*}$).

#### 3.1.3 Proliferation rate

1.3 × 10^5^ cells/h proliferate in compartment 3 ($\lambda_{3}\;n_{3}^{*}$).

We have also computed the stringency of thymic selection, which we define as given by the ratio:

$$\frac{\varphi_{3}\;n_{3}^{*}}{\phi }=6.79\%\;.$$

Finally, we have computed the (per cell) probability to die, given that the cell is in compartment *i* (*i* = 1, 2, 3), as well as the (per cell) probability to proliferate in the medulla. We have obtained:

$$\begin{array}{llll} & p_{1}=\frac{\mu_{1}}{\mu_{1}+\varphi_{1}}=65.8\%\;,\;\;p_{2}=\frac{\mu_{2}}{\mu_{2}+\varphi_{2}}=91.7\%\;,\; & & \;\\ & p_{3}=\frac{\mu_{3}}{\mu_{3}+\varphi_{3}+\lambda_{3}}=22.9\%\;,\;\;q_{3}=\frac{\lambda_{3}}{\mu_{3}+\varphi_{3}+\lambda_{3}}=42.2\%\;.\; & & \; \end{array}$$

### 3.2 Parameter estimation for the second model (means)

In this section, we make use of previously published experimental data (26) that provide thymocyte cell counts for the four subsets considered: pre-DPs, post-DPs, CD4 SPs, and CD8 SPs. We only make use of the experimental data for the wild type mice. The data will allow us to provide experimental estimates for the steady state thymocyte cell counts: $n_{1}^{*},n_{2}^{*},n_{4}^{*},n_{8}^{*}$. As described in Section 3.1, we also need residence times for each population subset, *τ_i_*, with *i* = 1, 2, 4, 8. If we make use of the model (see Section 2.2), the residence time in compartment *i* can be expressed as:

$$\begin{array}{llll} & \tau_{i}=\frac{1}{\varphi_{i}+\mu_{i}}\;,\;\mathrm{for}\;i\in \left\{1,2\right\}\;,\;\mathrm{and}\; & & \;\\ & \tau_{i}=\frac{1}{\xi_{i}+\mu_{i}}\;,\;\mathrm{for}\;i\in \left\{4,8\right\}\;.\; & & \; \end{array}$$

Recent experimental data provide support for asymmetric death rates in the CD4 and CD8 SP compartments (21). The estimated death rates for CD4 and CD8 SP thymocytes are^1^ *μ*_4_ = 0.04 day^−1^ and *μ*_8_ = 0.11 day^−1^. We also make use of the estimates derived in Section 3.1 for *ϕ, μ*_1_, *μ*_2_, *φ*_1_, and *φ*_2_. Finally, the average residence times in each compartment, as described in Section 3.1, are given by:

$$\begin{array}{llll} & \tau_{1}=60\;h=2.5\;\mathrm{days}\;,\;\tau_{2}=16\;h=0.67\;\mathrm{days}\;,\; & & \;\\ & \tau_{4}=96\;h=4\;\mathrm{days}\;,\;\tau_{8}=96\;h=4\;\mathrm{days}\;.\; & & \; \end{array}$$

**Figure 4:**
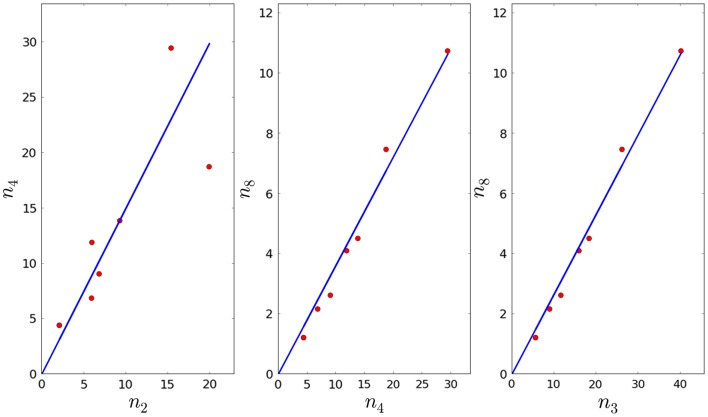
**Linear regression plots for the second model**.

In order to derive estimates for the model parameters, we have carried out the following steps:

1. Given *τ*_4_, *μ*_4_, and the fact that $\tau_{4}=\frac{1}{\mu_{4}+\xi_{4}}\;,$ we can obtain an estimate for *ξ*_4_.
2. In the same way, given *τ*_8_, *μ*_8_, and the fact that $\tau_{8}=\frac{1}{\mu_{8}+\xi_{8}}\;,$ we can obtain an estimate for *ξ*_8_.
3. We are now left with four remaining parameters: *φ*_4_, *φ*_8_, *λ*_4_, and *λ*_8_. We know that *φ*_2_ = *φ*_4_ + *φ*_8_. We sample *φ*_4_ in the interval [0, *φ*_2_], where *φ*_2_ is the mean value of the interval obtained in Section 2.1, and for each fiducial value for *φ*_4_, we compute the corresponding value for *φ*_8_.
4. Given *τ*_4_, *φ*_4_, and the fact that $n_{4}^{*}=\frac{n_{2}^{*}\varphi_{4}}{\tau_{4}^{-1}-\lambda_{4}}\;,$ we can compute the fraction $a_{3}=\frac{n_{2}^{*}}{n_{4}^{*}}$ by linear regression (see Figure 4), and thus obtain an estimate for *λ*_4_. Note that we will reject values of *λ*_4_ that imply the proliferation time is larger than 7 days (see Section 3.1).
5. In Section 3.1, we obtained an estimate for the mean of *λ*_3_, and we know that $\lambda_{4}\;n_{4}^{*}+\lambda_{8}\;n_{8}^{*}=\lambda_{3}\;n_{3}^{*}\;.$ As before, we can compute the fractions $a_{4}=\frac{n_{4}^{*}}{n_{8}^{*}}$ and $a_{5}=\frac{n_{3}^{*}}{n_{8}^{*}}$ by linear regression (see Figure 4), and thus obtain an estimate for *λ*_8_. Note that we will reject values of *λ*_8_ that imply the proliferation time is larger than 7 days (see Section 3.1). We note that the *p*-values for *a*_3_, *a*_4_, and *a*_5_ are given by 8.43 × 10^−3^, 3.33 × 10^−7^, and 4.56 × 10^−8^, respectively (smaller than the significance level).
6. From steps 3, 4, and 5 above, we have generated (a table of) values for *φ*_8_, *λ*_4_, and *λ*_8_, given a fiducial value for *φ*_4_ in the interval [0, *φ*_2_]. As discussed in Section 3.1, we only accept parameter values which provide thymocyte cell counts at time *t* = 21 days that are within one standard deviation from the experimentally determined values (see Table 1).

We obtain the following parameter values:

$$\begin{array}{llll} & \mu_{4}=0.04\;{\mathrm{day}}^{-1}\;,\;\;\mu_{8}=0.11\;{\mathrm{day}}^{-1}\;,\; & & \;\\ & \xi_{4}=0.21\;{\mathrm{day}}^{-1}\;,\;\;\xi_{8}=0.14\;{\mathrm{day}}^{-1}\;,\; & & \; \end{array}$$

and

$$\begin{array}{llll} & \varphi_{4}=0.070\;{\mathrm{day}}^{-1}\;,\;\;\varphi_{8}=0.054\;{\mathrm{day}}^{-1}\;,\; & & \;\\ & \lambda_{4}=0.216\;{\mathrm{day}}^{-1}\;,\;\;\lambda_{8}=0.093\;{\mathrm{day}}^{-1}\;.\; & & \; \end{array}$$

These parameters imply the following thymic selection rates:

#### 3.2.1 Death rates

2.05 × 10^4^ cells/h die by negative selection in compartment 4 ($\mu_{4}\;n_{4}^{*}$) and 1.95 × 10^4^ cells/h die by negative selection in compartment 8 ($\mu_{8}\;n_{8}^{*}$).

#### 3.2.2 Differentiation rates

2.50 × 10^4^ cells/h are CD4 positively selected in compartment 2, that is, become CD4 SP from post-DP ($\varphi_{4}\;n_{2}^{*}$), 1.90 × 10^4^ cells/h are CD8 positively selected in compartment 2, that is, become CD8 SP from post-DP ($\varphi_{8}\;n_{2}^{*}$), 1.08 × 10^5^ cells/h leave compartment 4 to go to the periphery ($\xi_{4}\;n_{4}^{*}$), and 2.48 × 10^4^ cells/h leave compartment 8 to go to the periphery ($\xi_{8}\;n_{8}^{*}$).

#### 3.2.3 Proliferation rates

11.10 × 10^4^ cells/h proliferate in compartment 4 ($\lambda_{4}\;n_{4}^{*}$) and 1.60 × 10^4^ cells/h proliferate in compartment 8 ($\lambda_{8}\;n_{8}^{*}$).

We can compute the stringency of thymic selection, defined by the ratio:

$$\frac{\xi_{4}\;n_{4}^{*}+\xi_{8}\;n_{8}^{*}}{\phi }=8.96\%\;.$$

We can provide an estimate for the cortical positive selection probabilities, that is the (per post-DP cell) probability to become a CD4 SP or a CD8 SP, and the probability to be negatively selected in the cortex. We have obtained:

$$\begin{array}{llll} & s_{4}=\frac{\varphi_{4}}{\mu_{2}+\varphi_{4}+\varphi_{8}}=4.7\%\;,\;\;s_{8}=\frac{\varphi_{8}}{\mu_{2}+\varphi_{4}+\varphi_{8}}=3.6\%\;,\; & & \;\\ & p_{2}=\frac{\mu_{2}}{\mu_{2}+\varphi_{4}+\varphi_{8}}=91.7\%\;.\; & & \; \end{array}$$

Finally, we have computed the (per cell) probability to die, given that the cell is in compartment *i*, as well as the (per cell) probability to proliferate in the medulla. We obtain:

$$\begin{array}{llll} p_{4} & =\frac{\mu_{4}}{\mu_{4}+\xi_{4}+\lambda_{4}}=8.6\%\;,\;\;q_{4}=\frac{\lambda_{4}}{\mu_{4}+\xi_{4}+\lambda_{4}}=46.3\%\;,\; & & \;\\ p_{8} & =\frac{\mu_{8}}{\mu_{8}+\xi_{8}+\lambda_{8}}=32.1\%\;,\;\;q_{8}=\frac{\lambda_{8}}{\mu_{8}+\xi_{8}+\lambda_{8}}=27.0\%\;.\; & & \; \end{array}$$

These probabilities imply that the probability to exit the thymus as a mature CD4 thymocyte (that has already reached the medulla) is given by $\frac{100-\left(8.6+46.3\right)}{100}$, which is 45.1%, and the probability to exit as a mature CD8 thymocyte (that has already reached the medulla) is given by $\frac{100-\left(32.1+27.0\right)}{100}$, which is 40.9%.

### 3.3 Sensitivity analysis

In this section, we explore the sensitivity of the parameters to perturbations in the experimental data. For the first model, the experimental data are given in terms of the following eight quantities:

$$\theta =\left(\tau_{1},\tau_{2},\tau_{3},\phi_{\mathrm{out}},a_{1},a_{2},{\bar{n}}_{3},{\bar{n}}_{1}\right)\;,$$

where *a*_1_, *a*_2_ are the coefficients of the linear regression of $\frac{n_{2}^{*}}{n_{1}^{*}}$ and $\frac{n_{3}^{*}}{n_{2}^{*}}$, respectively, and ${\bar{n}}_{i}$ is the experimental mean value of *n_i_*.

We perturb each entry of the vector *θ* by adding and subtracting 10% of its value. Therefore, we now have two values for *θ_i_*, equal to $\theta_{i}+\frac{1}{10}\theta_{i}$ and $\theta_{i}-\frac{1}{10}\theta_{i}$. Consequently, we have 2^8^ sets of *θ*, which will be used to compute the corresponding model parameters as described in Section 3.1. Parameter values will only be accepted if they provide a stable solution before *t* = 21 days.

For the second model, the experimental data is given in terms of the following seven quantities:

$$\theta =\left(\tau_{4},\tau_{8},\mu_{4},\mu_{8},a_{3},a_{4},a_{5}\right)\;,$$

where *a*_3_, *a*_4_, *a*_5_ are the coefficients of the linear regression of $\frac{n_{2}^{*}}{n_{4}^{*}}$, $\frac{n_{4}^{*}}{n_{8}^{*}}$, and $\frac{n_{3}^{*}}{n_{8}^{*}}$, respectively. We have made use of the means of the following parameters of the first model: *ϕ, φ*_1_, *μ*_1_, *φ*_2_, *μ*_2_, *λ*_3_. We perturb each entry of the vector *θ* as described above. Therefore, we have $2^{7}n_{\varphi_{4}}$ sets of *θ*, with $n_{\varphi_{4}},$ the number of different values considered for *φ*_4_ in the interval (0, *φ*_2_). Parameter values will only be accepted if they provide a stable solution before *t* = 21 days.

The results of the sensitivity analysis, with 95% trimmed intervals^2^ and minimum–maximum interval ranges, are given in Table 2.

**Table 2:** **Means, 95% trimmed and minimum–maximum intervals of the model parameters**.

Parameter	Mean value	95% Trimmed interval	Minimum–maximum interval range
*ϕ*	35.86 × 10^6^ cells/day	(35.65 × 10^6^, 35.07 × 10^6^) cells/day	(28.93, 43.21 × 10^6^) cells/day
*φ*_1_	0.139 day^−1^	(0.138, 0.140) day^−1^	(0.112, 0.167) day^−1^
*φ*_2_	0.136 day^−1^	(0.134, 0.139) day^−1^	(0.041, 0.274) day^−1^
*φ*_4_	0.140 day^−1^	(0.136, 0.145) day^−1^	(0.060, 0.264) day^−1^
*φ*_8_	0.134 day^−1^	(0.129, 0.138) day^−1^	(0.010, 0.214) day^−1^
*μ*_1_	0.265 day^−1^	(0.263, 0.267) day^−1^	(0.196, 0.333) day^−1^
*μ*_2_	1.372 day^−1^	(1.365, 1.378) day^−1^	(1.083, 1.618) day^−1^
*μ*_4_	0.040 day^−1^	*n*/*a*	(0.036, 0.044) day^−1^
*μ*_8_	0.110 day^−1^	*n*/*a*	(0.099, 0.121) day^−1^
*λ*_4_	0.181 day^−1^	(0.179, 0.184) day^−1^	(0.116, 0.226) day^−1^
*λ*_8_	0.085 day^−1^	(0.080, 0.090) day^−1^	(0.078, 0.092) day^−1^
*ξ*_4_	0.231 day^−1^	(0.230, 0.233) day^−1^	(0.229, 0.233) day^−1^
*ξ*_8_	0.152 day^−1^	(0.150, 0.154) day^−1^	(0.149, 0.155) day^−1^

### 3.4 Variability in the selection rates

The (trimmed and minimum–maximum) intervals derived in Section 3.3 allow us to estimate the variability in the different selection rates discussed in Sections 3.1 and 3.2. For example, given variations in the parameters, the corresponding variations in the selection rates can be shown to be:

(10) $$\begin{array}{ll} \Delta p_{i} & =\frac{1}{{\left(\mu_{i}+\varphi_{i}\right)}^{2}}\left(\varphi_{i}\Delta \mu_{i}+\mu_{i}\Delta \varphi_{i}\right)\;\mathrm{for}\;i=1,2\;, \end{array}$$

(11) $$\begin{array}{ll} \Delta p_{3} & =\frac{1}{{\left(\mu_{3}+\varphi_{3}+\lambda_{3}\right)}^{2}}\left[\left(\varphi_{3}+\lambda_{3}\right)\Delta \mu_{3}+\mu_{3}\Delta \varphi_{3}+\mu_{3}\Delta \lambda_{3}\right]\;, \end{array}$$

(12) $$\begin{array}{ll} \Delta q_{3} & =\frac{1}{{\left(\mu_{3}+\varphi_{3}+\lambda_{3}\right)}^{2}}\left[\lambda_{3}\Delta \mu_{3}+\lambda_{3}\Delta \varphi_{3}+\left(\mu_{3}+\varphi_{3}\right)\Delta \lambda_{3}\right]\;, \end{array}$$

(13) $$\begin{array}{l} \Delta s_{i}=\frac{1}{{\left(\mu_{2}+\varphi_{i}+\varphi_{j}\right)}^{2}}[\varphi_{i}\Delta \mu_{2}+\varphi_{i}\Delta \varphi_{j}\\ \;+(\mu_{2}+\varphi_{j})\Delta \varphi_{i}]\text{ for }i=4,j=8\text{ or }i=8,\;j=4, \end{array}$$

(14) $$\begin{array}{l} \Delta p_{i}=\frac{1}{{\left(\mu_{i}+\xi_{i}+\lambda_{i}\right)}^{2}}[\mu_{i}\Delta \xi_{i}+\mu_{i}\Delta \lambda_{i}\\ \;+(\xi_{i}+\lambda_{i})\Delta \mu_{i}]\text{ for }i=4,8, \end{array}$$

(15) $$\begin{array}{l} \Delta q_{i}=\frac{1}{{\left(\mu_{i}+\xi_{i}+\lambda_{i}\right)}^{2}}[\lambda_{i}\Delta \xi_{i}+\lambda_{i}\Delta \mu_{i}\\ \;+(\xi_{i}+\mu_{i})\;\Delta \;\lambda_{i}]\text{ for }i=4,8. \end{array}$$

We present in Table 3 the variability of the selection rates.

**Table 3:** **Selection rate values (initial and after perturbation) and their variability intervals**.

Rate	Initial value (%)	After perturbation (%)	Δ Value (%)	Δ Min–max (%)
*p*_1_	65.8	65.66	±0.76	±20.55
*p*_2_	91.7	90.98	±0.49	±17.28
*p*_3_	22.91	24.07	±0.90	±28.22
*q*_3_	42.24	41.74	±0.99	±30.53
*s*_4_	4.69	8.51	±0.80	±15.16
*s*_8_	3.61	8.13	±0.79	±15.03
*p*_4_	8.59	8.84	±0.37	±4.91
*q*_4_	46.29	40.07	±1.29	±20.49
*p*_8_	32.12	31.71	±2.25	±35.37
*q*_8_	27.0	24.5	±3.58	±64.81

## 4 Discussion

We have brought together experimental data with a mathematical compartment model [similar to other progression models of CD4 and CD8 T cell development (13, 14, 18, 21, 33)] to provide estimates for the selection events that take place in the thymus. We have made use of a range of experimental data: (i) steady state thymocyte cell counts (26), mean residence times in each compartment (27–29), murine thymic export rate (14, 26, 30), and recently reported asymmetric death rates for the CD4 SP and CD8 SP thymocytes (21). Our preliminary results support the unexpectedly high death rate in the post-DP thymocyte population observed in Ref. (21). We note that our approach is unrelated to that of Sinclair et al. both experimentally and mathematically (21). This rate, *μ*_2_, has been estimated to be at least an order of magnitude larger than any of the other death rates in the pre-DP, CD4 SP, or CD8 SP pools (see Table 2). In terms of selection rates, our analysis yields the following: pre-selection thymocytes (pre-DPs) have a 65.8% probability of dying by neglect in the cortex, and a 34.2% probability of becoming post-selection thymocytes (post-DPs). At the post-selection stage, post-DPs have a 91.7% probability of dying by negative selection (apoptosis) in the cortex, a 4.7% probability of becoming CD4 SPs, and a 3.6% probability of becoming CD8 SPs. In the medulla, CD4 SPs have an 8.6% probability of dying by negative selection (apoptosis), whereas CD8 SPs have a 32.1% probability of dying by negative selection. CD4 SPs have a 45.1% probability of exiting the thymus and reaching the periphery as mature thymocytes, whereas that probability for CD8 SPs is only 40.9%. Finally, the data supports some level of cellular proliferation in the medulla, with CD4 SPs having a 46.3% probability of proliferation and CD8 SPs a 27% probability.

Earlier work by Mehr and collaborators combined experimental and theoretical approaches to estimate thymic selection rates (13, 33), neglected death rates in the medulla, but considered potential feedback from mature T cells. In agreement with these authors, our results indicate that thymocyte death is highest at the post-DP stage. However, as death in the medulla had been neglected, these authors concluded that the CD4:CD8 ratio in SP thymocytes is determined by the differentiation rates. In this paper, we have made use of CD4 and CD8, or medullary, death rates, which allowed us to directly compare cortical (DP) to medullary (SP) death rates. Furthermore, our approach allowed us to conclude that medullary, or SP, death was due to negative selection, as it was rescued by Bim deficiency (26). Sinclair et al. also recently addressed the temporal dynamics of thymic selection using an unrelated approach (both experimentally and mathematically) (21). While their experimental approach did not allow them to distinguish death by negative selection from death by other mechanisms, their overall finding was consistent with ours, that thymocyte death is highest at the post-DP stage.

Further attempts to quantify thymic selection rates making use of mathematical models also include those of Faro et al. (12). The mathematical model developed by Faro and collaborators does not include time dynamics, but describes the relationship between the number of selecting ligands and the probability of selection of a given thymocyte. Thomas-Vaslin et al. (14) obtained estimates of thymic selection rates, using an experimental procedure that temporarily blocks thymic output and a mathematical model in which rates of transit from compartment to compartment depend on the number of cell divisions. Their model can capture the thymic “conveyor belt” (34, 35) scheme, but requires more differential equations and more parameters than equation (6). Despite the differences between their theoretical and experimental models and ours, similar estimates for thymic selection rates are found. For example, we estimate that 1.2 million post-DP become CD4 SP thymocytes per day and 0.5 million post-DP become CD8 SP thymocytes per day; their estimates are 0.9 and 0.2 million, respectively. Finally, we estimate that 2.6 million CD4 SP thymocytes per day and 0.6 million CD8 SP thymocytes per day exit the thymus. Their estimates are 2.4 and 0.5 million, respectively.

Our estimates of how many CD4 and CD8 SP thymocytes survive and exit the thymus reflect the skewed CD4:CD8 SP thymocyte ratio observed in C57BL/6 mice, which is approximately 4:1 (36). This ratio is similar to the reported CD4:CD8 ratio of recent thymic emigrants (37), and raises the question of what accounts for the CD4 bias. While we were able to determine death and differentiation rates for both CD4 and CD8 SP thymocytes (see Table 2), our experimental approach did not allow us to determine what fraction of the post-DP pool was MHC class I versus II restricted. Therefore, we could not address the issue of when and how the CD4:CD8 bias becomes established. The approach of Sinclair et al., which used MHC class I and class II deficiency, allowed them to address this question. Their data suggest that the skewed CD4:CD8 ratio reflects asymmetry in post-selection DP death rates, rather than more efficient positive selection of CD4 compared to CD8 thymocytes (21). Yet, the parameter estimation allows us to compare the following different CD4:CD8 ratios (see Section 3.2): (i) the CD4:CD8 ratio of positive selection in the post-DP pool (differentiation from post-DP to either CD4 SP or CD8 SP) is given by $\frac{\varphi_{4}}{\varphi_{8}}\approx 5:4$, (ii) the CD4:CD8 ratio in the SP pool is given by $\frac{n_{4}^{*}}{n_{8}^{*}}\approx 3:1$, and (iii) the CD4:CD8 ratio of positive selection in the SP pool (differentiation from SP to peripheral early thymic emigrants) is given by $\frac{\xi_{4}n_{4}^{*}}{\xi_{8}n_{8}^{*}}\approx 4:1$. Our observations indicate that the CD4 bias is progressively established, as the thymocytes mature from the post-DP stage until the exit of the SP stage to migrate to the periphery.

Our mathematical analysis has also allowed us to estimate the stringency of thymic selection, defined by:

$$\sigma =\frac{\xi_{4}\;n_{4}^{*}+\xi_{8}\;n_{8}^{*}}{\phi }=8.96\%\;,$$

that is, the ratio between the number of thymocytes per unit time that exit the thymus and the number of thymocytes per unit time that enter the pre-DP stage. The sensitivity analysis described in Section 3.3 allows us to provide a value of Δ*σ* = 0.2%, where we have made use of the minimum–maximum interval ranges (see fourth column of Table 2). A different measure of stringency could be based on the probability of a cell surviving the maturation process. In our notation, this would correspond to the following:

$$\left(1-p_{1}\right)\times \left(1-p_{2}\right)\times \left(1-p_{3}\right)=2.19\%\;.$$

We note that this measure of stringency is the probability of not dying in any of the three compartments considered in the model (pre-DP, post-DP, and SP). As discussed in Appendix A.1, and given that in the SP pool, thymocytes may proliferate, there is a need to consider this special case. Our estimates suggest that a population of 10^3^ pre-DP thymocytes will yield 69 CD4 and 25 CD8 SP thymocytes that leave the medulla to get incorporated into the peripheral naive T cell pool (see details in Appendix A.1).

**Figure 5:**
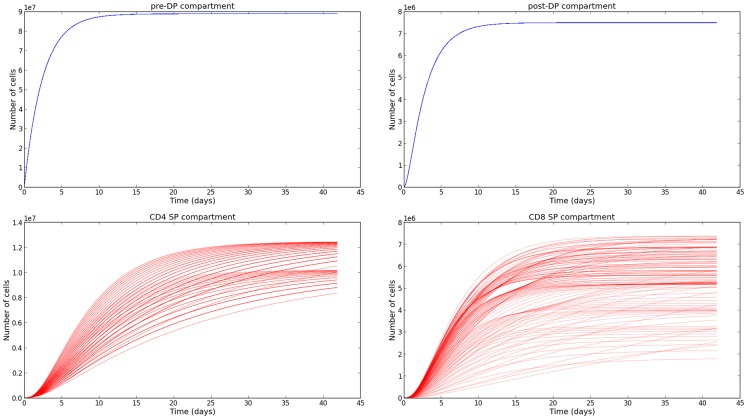
**Time evolution of the thymocyte populations in the second model**. The different trajectories correspond to the parameter values and ranges described in Table 2.

The sensitivity analysis (see Section 3.3) and the variability of the selection rates derived from it (see Section 3.4) give us the confidence to conclude, that our parameter estimation is robust. We are aware that the experimental data we have made use of [steady state thymocyte cell counts (26)] do not provide the exquisite time resolution described in Ref. (21). However, the supporting mathematical model described in Section 2.2, allows us to obtain the time evolution of the thymocyte populations, once the parameters have been estimated. In Figure 5, we plot the time evolution of the total number of cells in each compartment of the mathematical model: pre-DP, post-DP, CD4 SP, and CD8 SP thymocytes. We start with no cells at time zero, *n_i_*(*t* = 0) = 0 for *i* = 1, 2, 4, 8. Trajectories have been plotted for a period of 6 weeks and have been computed for every permutation of the parameter set presented in Table 2. The subset of parameters shared with the simple model (*ϕ, φ*_1_, *μ*_1_, *μ*_2_), were fixed at their mean values. Thus, 548 distinct parameter sets were generated. The system of equations (6) was solved using a fourth order Runge–Kutta method (Python source code).

The approaches introduced in this paper have shed some light on the probabilities and timescales that characterize cellular fate in the thymus after the DN stage. We plan to generalize the mathematical model introduced here, making use of experimental data for the strength of TCR binding in Nur77^GFP^ mice (26), to investigate issues such as the death rate in the post-DP pool and the CD4:CD8 ratio. Our model assumes that all progenitors in a particular pool behave with identical kinetics, i.e., move through the various stages of selection at the same rate. Future model refinements will come from consideration of the heterogeneity of the pools, which are known to include cells that will become iNKT cells, regulatory T cells, and intra-epithelial lymphocytes (2). It is also possible that progenitors of the same general class move through the selection process with different kinetics (34). The models introduced here can serve as a first step to study human thymic selection, although comprehensive data on human thymic subsets, their sizes, and residence times are not yet available. It would be of great interest to apply the model to data on thymic subsets and cellularity in children, keeping in mind that residence times of human subsets may differ from murine ones (38). Finally, we note that we have not mentioned the relevance of cytokines, such as IL-7, during thymic development. Some differences have already been described for the role of IL-7R in human versus mouse T cell development (38, 39). We hope in the near future to combine mechanistic mathematical models of IL-7 and IL-7R (40) with the T cell development model introduced here to address these issues.

## Conflict of Interest Statement

The authors declare that the research was conducted in the absence of any commercial or financial relationships that could be construed as a potential conflict of interest.

## Footnotes

^1^Private communication from Ben Seddon and Andy Yates.

^2^We define the 95% trimmed interval to be the result of the sensitivity analysis after trimming the lower and upper 2.5% of values.

## Appendix

### A.1 Stringency of thymic selection: A stochastic model

In this section, we present the details that allow us to compute the stringency of thymic selection for the mathematical model considered in Section 2.2.

Let us assume that at time *t* = 0, there exists a single T cell in a given compartment. In our case, the compartment can be the pre-DP, post-DP, or SP (either CD4 SP or CD8 SP) stages. The cell, at any time, may die (with rate, *μ*), divide (with rate, *λ*), and produce two daughter cells, or leave the compartment (with rate, *ξ*) to enter a different compartment. The waiting times for each event are assumed to be exponentially distributed, and daughter cells are assumed to behave identically to the initial single cell. We introduce the bivariate Markov process {*X*(*t*), *Y* (*t*)}*_t_*_≥0_, where *X*(*t*) is the number of cells in the compartment at time *t*, and *Y* (*t*) is the number of cells which have left the compartment (to enter a different compartment). Our aim is to calculate the expected number of cells (and variance) that leave the compartment.

State probabilities for the Markov process are defined as follows (41)

(A1) $$p_{\left(x,y\right)}\left(t\right)=\mathrm{Prob}\left\{X\left(t\right)=x,Y\left(t\right)=y|X\left(0\right)=1,Y\left(0\right)=0\right\}\;,$$

and transition probabilities for this process are defined as follows (41)

(A2) $$\begin{array}{ccc} & p_{\left(w,z\right),\left(x,y\right)}\left(\Delta t\right)=\mathrm{Prob}\{X\left(t+\Delta t\right)=w, & \\ & \;Y(t+\Delta t)=z|X(t)=x,Y(t)=y\}\\ & \;=\left\{\begin{array}{cc} \lambda x\Delta t+o\left(\Delta t\right),\; & \;\;\mathrm{if}\;w=x+1,z=y\;,\\ \mu x\Delta t+o\left(\Delta t\right),\; & \;\;\mathrm{if}\;w=x-1,z=y\;,\\ \xi x\Delta t+o\left(\Delta t\right),\; & \;\;\mathrm{if}\;w=x-1,z=y+1\;,\\ 1-\left(\lambda +\mu +\xi \right)x\Delta t+o\left(\Delta t\right),\; & \;\;\mathrm{if}\;w=x,z=y\;,\\ o\left(\Delta t\right),\; & \;\;\mathrm{if}\;w,z\;\mathrm{otherwise}\;.\\ \; \end{array}\right. \end{array}$$

The Kolmogorov (or master) equation for this process is given by (41)

(A3) $$\begin{array}{llll} \frac{dp_{\left(x,y\right)}\left(t\right)}{dt} & =\lambda \;\left(x-1\right)p_{\left(x-1,y\right)}\left(t\right)+\mu \;\left(x+1\right)p_{\left(x+1,y\right)}\left(t\right)\; & & \;\\ & \;+\xi \;\left(x+1\right)p_{\left(x+1,y-1\right)}\left(t\right)-\left(\lambda +\mu +\xi \right)\;x\;p_{\left(x,y\right)}\left(t\right)\;.\; \end{array}$$

Let *m_X_*(*t*) be the expected number of cells in the compartment under consideration, and *m_Y_*(*t*) be the expected number of cells which have left the compartment. Similarly, let *m_XX_*(*t*) be the expectation of the random variable *X*(*t*)^2^, *m_XY_*(*t*) be the expectation of the random variable *X*(*t*)*Y* (*t*), and *m_YY_*(*t*) be the expectation of the random variable *Y* (*t*)^2^. Then, making use of the probability generating function technique (41), we derive the time evolution for the first two moments of the system:

(A4) $$\begin{array}{ll} \frac{dm_{X}\left(t\right)}{dt} & =\left(\lambda -\mu -\xi \right)m_{X}\left(t\right)\;, \end{array}$$

(A5) $$\begin{array}{ll} \frac{dm_{Y}\left(t\right)}{dt} & =\xi m_{X}\left(t\right)\;, \end{array}$$

(A6) $$\begin{array}{ll} \frac{dm_{XX}\left(t\right)}{dt} & =2\left(\lambda -\mu -\xi \right)m_{XX}\left(t\right)+\left(\lambda -\mu -\xi \right)m_{X}\left(t\right)\;, \end{array}$$

(A7) $$\begin{array}{ll} \frac{dm_{XY}\left(t\right)}{dt} & =\left(\lambda -\mu -\xi \right)m_{XY}\left(t\right)+\xi \left[m_{XX}\left(t\right)-m_{X}\left(t\right)\right]\;, \end{array}$$

(A8) $$\begin{array}{ll} \frac{dm_{YY}\left(t\right)}{dt} & =\xi \left[2m_{XY}\left(t\right)+m_{X}\left(t\right)\right].\; \end{array}$$

Given that we start with a single cell, the expected number of cells at time *t* is given by

(A9) $$m_{X}\left(t\right)=e^{\left(\lambda -\mu -\xi \right)t}\;.$$

Under the restriction *λ* < *μ* + *ξ*, the expected number of cells tends to zero as *t* → +∞. This implies that all cells from the single T cell progenitor either die or leave the compartment for sufficiently large times. The expected number of cells which leave the compartment is given by

(A10) $$m_{Y}\left(t\right)=\frac{\xi }{\mu +\xi -\lambda }\left[1-e^{\left(\lambda -\mu -\xi \right)t}\right]\;.$$

As *t* → +∞, the expected number of cells to leave the compartment can be shown to be

(A11) $$\underset{t\to +\infty }{\lim}\;m_{Y}\left(t\right)=\frac{\xi }{\mu +\xi -\lambda }\;.$$

We now solve the remaining ODEs equations (A6–A8), to find

(A12) $$\begin{array}{llll} m_{YY}\left(t\right) & =\frac{2\lambda \xi^{2}}{{\left(\lambda -\mu -\xi \right)}^{3}}\left[\frac{1}{2}e^{2\left(\lambda -\mu -\xi \right)t}-e^{\left(\lambda -\mu -\xi \right)t}\right]\; & & \;\\ & \;-\frac{4\lambda \xi^{2}}{{\left(\lambda -\mu -\xi \right)}^{2}}\left(t-\frac{1}{\lambda -\mu -\xi }\right)e^{\left(\lambda -\mu -\xi \right)t}\; & & \;\\ & \;+\frac{\xi }{\lambda -\mu -\xi }e^{\left(\lambda -\mu -\xi \right)t}-\frac{2\lambda \xi^{2}}{{\left(\lambda -\mu -\xi \right)}^{3}}\; & & \;\\ & \;-\frac{\xi }{\lambda -\mu -\xi }\;. \end{array}$$

It, therefore, follows that the random variable *Y* (*t*), which represents the number of cells leaving the compartment under consideration, has the following variance (in the limit *t* → +∞)

(A13) $$\begin{array}{llll} \sigma_{Y}^{2} & =\underset{t\to \infty }{\lim}\;\left[m_{YY}\left(t\right)-m_{Y}{\left(t\right)}^{2}\right]=\frac{2\lambda \xi^{2}}{{\left(\mu +\xi -\lambda \right)}^{3}}+\frac{\xi }{\mu +\xi -\lambda }\; & & \;\\ & \;-\frac{\xi^{2}}{{\left(\mu +\xi -\lambda \right)}^{2}}\;. \end{array}$$

### A.2 Stringency of thymic selection in the four compartment model

The previous example can easily (but laboriously) be extended to the mathematical model introduced in Section 2.2.

We may evaluate the expected number of, for example, CD4^+^ T cells produced by a single pre-DP progenitor (or more generally *N* pre-DP progenitors). We only present the time evolution of the moment generating function. The deterministic equations describing the mean and variance of the numbers of cells in each compartment (pre-DP, post-DP, SP CD4, and SP CD8) are left for the reader to derive. When counting the number of CD4^+^ T cells leaving the thymus, the moment generating function satisfies the following partial differential equation

(A14) $$\begin{array}{llll} \frac{\mathrm{\partial M}}{\mathrm{\partial t}} & =\mu_{1}\left(e^{-\theta_{1}}-1\right)\frac{\mathrm{\partial M}}{\partial \theta_{1}}+\varphi_{1}\left(e^{-\theta_{1}}e^{\theta_{2}}-1\right)\frac{\mathrm{\partial M}}{\partial \theta_{1}}\; & & \;\\ & \;+\left(\mu_{2}+\varphi_{8}\right)\left(e^{-\theta_{2}}-1\right)\frac{\mathrm{\partial M}}{\partial \theta_{2}}+\varphi_{4}\left(e^{-\theta_{2}}e^{\theta_{4}}-1\right)\frac{\mathrm{\partial M}}{\partial \theta_{2}}\; & & \;\\ & \;+\mu_{4}\left(e^{-\theta_{4}}-1\right)\frac{\mathrm{\partial M}}{\partial \theta_{4}}+\lambda_{4}\left(e^{\theta_{4}}-1\right)\frac{\mathrm{\partial M}}{\partial \theta_{4}}\; & & \;\\ & \;+\xi_{4}\left(e^{-\theta_{4}}e^{\theta_{8}}-1\right)\frac{\mathrm{\partial M}}{\partial \theta_{4}}\;. \end{array}$$

The symmetry of the mathematical model implies that an equivalent equation for the number of CD8^+^ T cells leaving the thymus can be obtained by interchanging the indexes 4 and 8. For our derived parameter set, the previous equation allows us to conclude that the expected number of CD4^+^ T cells, a single thymocyte in the pre-DP compartment produces, is 0.069 (standard deviation 0.96), whereas the expected number of CD8^+^ T cells which leave the thymus is 0.025 (standard deviation 0.59).

To put this into perspective, a population of 10^3^ pre-DP thymocytes is expected to produce 69 CD4^+^ T cells which leave the thymus (standard deviation 66), and 25 CD8^+^ T cells (standard deviation 41). More generally, a population of *N* pre-DP thymocytes is expected to produce 0.069*N* CD4^+^ T cells and 0.025*N* CD8^+^ T cells.
